# Whole genome sequence and characterisation of *Streptococcus suis* 3112, isolated from snakeskin gourami, *Trichopodus pectoralis*

**DOI:** 10.1186/s12864-024-10736-x

**Published:** 2024-08-28

**Authors:** Pakorn Aiewsakun, Wuthiwat Ruangchai, Bharkbhoom Jaemsai, Thavin Bodharamik, Watcharachai Meemetta, Saengchan Senapin

**Affiliations:** 1 Department of Microbiology, Faculty of Science, Mahidol University, 272, Rama VI Road, Ratchathewi, Bangkok, 10400 Thailand; 2 Pornchai Matangkasombut Center for Microbial Genomics, Department of Microbiology, Faculty of Science, Mahidol University, Bangkok, 10400 Thailand; 3 Fish Health Platform, Center of Excellence for Shrimp Molecular Biology and Biotechnology (Centex Shrimp), Faculty of Science, Mahidol University, Bangkok, 10400 Thailand; 4 National Center for Genetic Engineering and Biotechnology (BIOTEC), National Science and Technology Development Agency (NSTDA), Pathum Thani, 12120 Thailand

**Keywords:** *Streptococcus suis*, Whole genome, Cross-species transmission, Fish pathogen

## Abstract

**Background:**

*Streptococcus suis* (*S. suis*) is an important swine and human pathogen. A recent study reported the first isolate of *S. suis* capable of infecting fish, designated as *S. suis* strain 3112. The bacterium was isolated from snakeskin gourami (*Trichopodus pectoralis*), an economically important fish species native to Southeast Asia, and it was previously shown that it can infect and cause lethal streptococcosis in the fish.

**Results:**

In this study, we present the complete genome of *S. suis* 3112. Molecular sequence analysis revealed that it belongs to serotype 6, sequence type 2340. Phylogenetic analysis showed that the bacterium clustered with healthy-pig *S. suis* isolates, suggestive of an ultimate swine (as opposed to human) origin of the bacterium. Two fluoroquinolone resistance genes are present in the bacterial genome, namely *patA* and *patB.* Our results showed that both genes are expressed in our bacterium, and the bacterium is resistant to norfloxacin, but is still sensitive to other fluoroquinolones, including ciprofloxacin, enrofloxacin, and sparfloxacin. Additionally, the bacterium is sensitive to β-lactams, tetracyclines, sulphonamides, and an aminoglycoside.

**Conclusions:**

This study reports and describes the complete genome of *S. suis* 3112, the first isolate of *S. suis* known to infect fish, and provides further insights into the bacterial isolate, particularly regarding its drug resistance profile. These results will facilitate further investigations of the comparative genomics and pathogenic characteristics of *S. suis*, as well as the development of control strategies against this newly-identified fish pathogen.

**Supplementary Information:**

The online version contains supplementary material available at 10.1186/s12864-024-10736-x.

## Background

*Streptococcus suis* (*S. suis*) is an important Gram-positive swine and human bacterial pathogen [1–3]. In pigs, *S. suis* infections can cause septicaemia, arthritis, and pneumonia, leading to acute death, particularly in piglets, resulting in significant economic losses for the pork industry in the past [3, 4]. In humans, *S. suis* infections usually occur in individuals with occupational exposure to swine, such as farmers, veterinarians, and meat processing workers. Common symptoms of human infections include meningitis, sepsis, septic arthritis, endocarditis, toxic shock syndrome, endophthalmitis, and hearing loss [5].

In addition to its main hosts pigs and humans, *S. suis* has been reported to infect birds [6, 7] and various other mammals, including ruminants, cats, dogs, deer, horses, and rabbits [8, 9]. Recently, a study reported the first documented case of *S. suis* causing lethal streptococcosis in snakeskin gourami (*Trichopodus pectoralis*), named *S. suis* strain 3112, thereby expanding the potential host range of this bacterium [10]. Experimental infections revealed that the infection fatality rates of the bacterium ranged from 50 to 92.5% in snakeskin gourami, depending on the infection dose and fish size [10]. Infected fish displayed several clinical symptoms, including skin haemorrhage, exophthalmos (eye protrusion), and erratic swimming, closely resembling naturally diseased fish [10]. Snakeskin gourami is an economically important fish species native to Southeast Asia. Although further study is required to fully assess the socioeconomic impact of this bacterium on fish aquaculture, previous findings suggested that it should be closely monitored as a potential emerging fish pathogen.

In this study, we conducted whole genome sequencing and characterisation of *S. suis* 3112. Our analysis revealed that this bacterium belongs to serotype 6, sequence type (ST) 2340. Furthermore, our results suggest that *S. suis* 3112 likely emerged through cross-species transmission ultimately from pigs to fish. We showed that the bacterium’s genome harbours and expresses fluoroquinolone resistance genes, namely *patA* and *patB*, and it is resistant to norfloxacin, but is still sensitive to other fluoroquinolones, including ciprofloxacin, enrofloxacin, and sparfloxacin, as well as β-lactams, tetracyclines, sulphonamides, and an aminoglycoside. These results further our knowledge and understanding of this newly-identified fish pathogen.

## Results and discussion

### *S. suis* 3112 whole genome

**Fig. 1:**
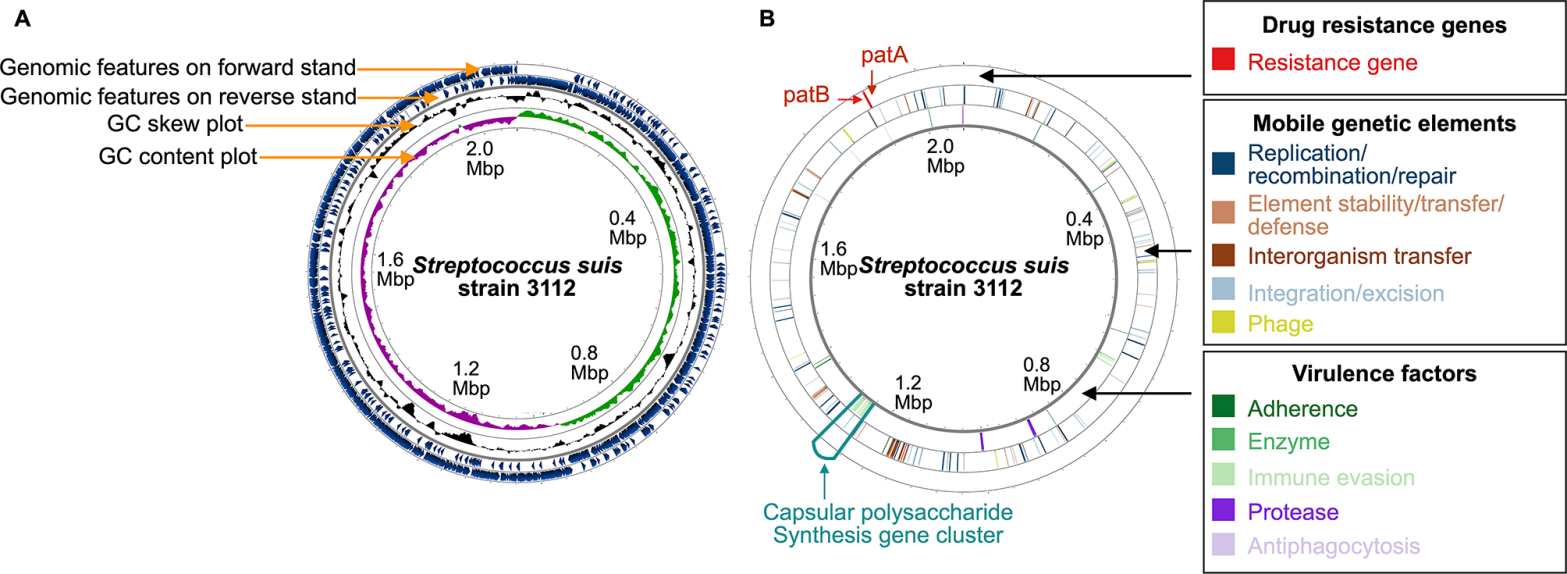
Overview of *S. suis* 3112 whole genome (**A**), and drug resistance genes, mobile genetic elements, and virulence factors identified in the bacterial genome (**B**)

The genome of *S. suis* 3112 was sequenced using both the 2nd generation (Illumina) and 3rd generation (Oxford nanopore) sequencing technologies (Table 1). A total of 2,586,035 paired-end short reads (SRR24927969: 713,395,771 bases), and 61,261 long reads (SRR24941354: 227,332,593 bases) were obtained after read cleaning. From these clean reads, the entire bacterial genome was successfully assembled into a single circular chromosome, and no plasmids were found (Table 2, and Fig. 1). The chromosome assembly has a length of 2,075,993 bases, and has an average short-read and long-read assembly depth of 365.11× and 67.79×, respectively. 95% of the chromosome positions have at least 253× short-read and 47× long-read assembly depths, supporting that most of the chromosomal regions were well-assembled. The chromosome has a GC content of 41.35%, and contains a total of 2,001 genomic features, including 1,861 coding genes, 72 RNA genes (4 5 S rRNA genes, 4 16 S rRNA genes, 4 23 S rRNA gene, 56 tRNA genes, and 4 ncRNA genes), and 68 pseudogenes. VFanalyzer [11] detected 39 virulence factors involved in 5 distinct biological functions (Supplementary Table 1), and mobileOG-db [12] detected 159 mobile genetic elements (Supplementary Table 2) scattering across the genome, divided into 5 classes (39 elements mediating replication/recombination/repair; 70 elements mediating sequence integration/excision; 26 elements mediating interorganism genetic material transfer; 16 elements mediating bacteria stability/transfer/defence, and 8 phage-like elements). Two drug resistance genes, *patA* and *patB*, were also detected in the genome by using Resistance Gene Identifier with the Comprehensive Antibiotic Resistance Database (CARD RGI) [13], known to confer resistance to fluoroquinolone antibiotics [14–16].

**Table 1:** Summary statistics of raw sequencing data

Name	Sequencing platform	Sequencing layout	Number of spots	Number of bases	Avg. length (bases)		SD. length (bases)		GC content (%)
					For.	Rev.	For.	Rev.	
**Short-read sequencing**									
SRR24927969	ILLUMINA (NovaSeq 6000)	PAIRED	3,836,892	888.7 M	125	106	43.1	49.8	42.5
***Long-read sequencing***									
SRR24941354	OXFORD NANOPORE (MinION)	SINGLE	61,262	232.6 M	3797		3442.8		41.9

**Table 2:** Assembly and annotation summary statistics

**Assembly statistics**	
GenBank accession number	CP097577.2
Assembly program	Unicycler V.0.4.8
Genome size	2.1 Mb
Total ungapped length	2.1 Mb
Number of chromosomes	1
Number of scaffolds	1
Scaffold N50	2.1 Mb
Scaffold L50	1
Number of contigs	1
Contig N50	2.1 Mb
Contig L50	1
GC percent	41
Genome coverage	Short-read: 365.11×; long-read: 67.79×
Assembly level	Complete Genome
***Annotation information***	
Annotation program	NCBI Prokaryotic Genome Annotation Pipeline (PGAP) v.6.1
Genes	2,001
Protein-coding	1,861

### *S. suis* 3112 serotyping and its *cps* locus

*S. suis* was originally classified into 35 serotypes based on the antigenicity of the bacterial capsular polysaccharide (CPS), namely serotype 1 to 34 and 1/2 [17–20]. Some of these serotypes have later been proposed to be re-classified as a new bacterial species [21], and additional CPS serotypes have also been proposed [22–25]. However, many diagnostic laboratories still use the original 35-serotype scheme for *S. suis* identification, and among these, serotype 2 is recognised as the most common cause of infections in piglets worldwide and a major agent of zoonotic infections [26].

**Fig. 2:**
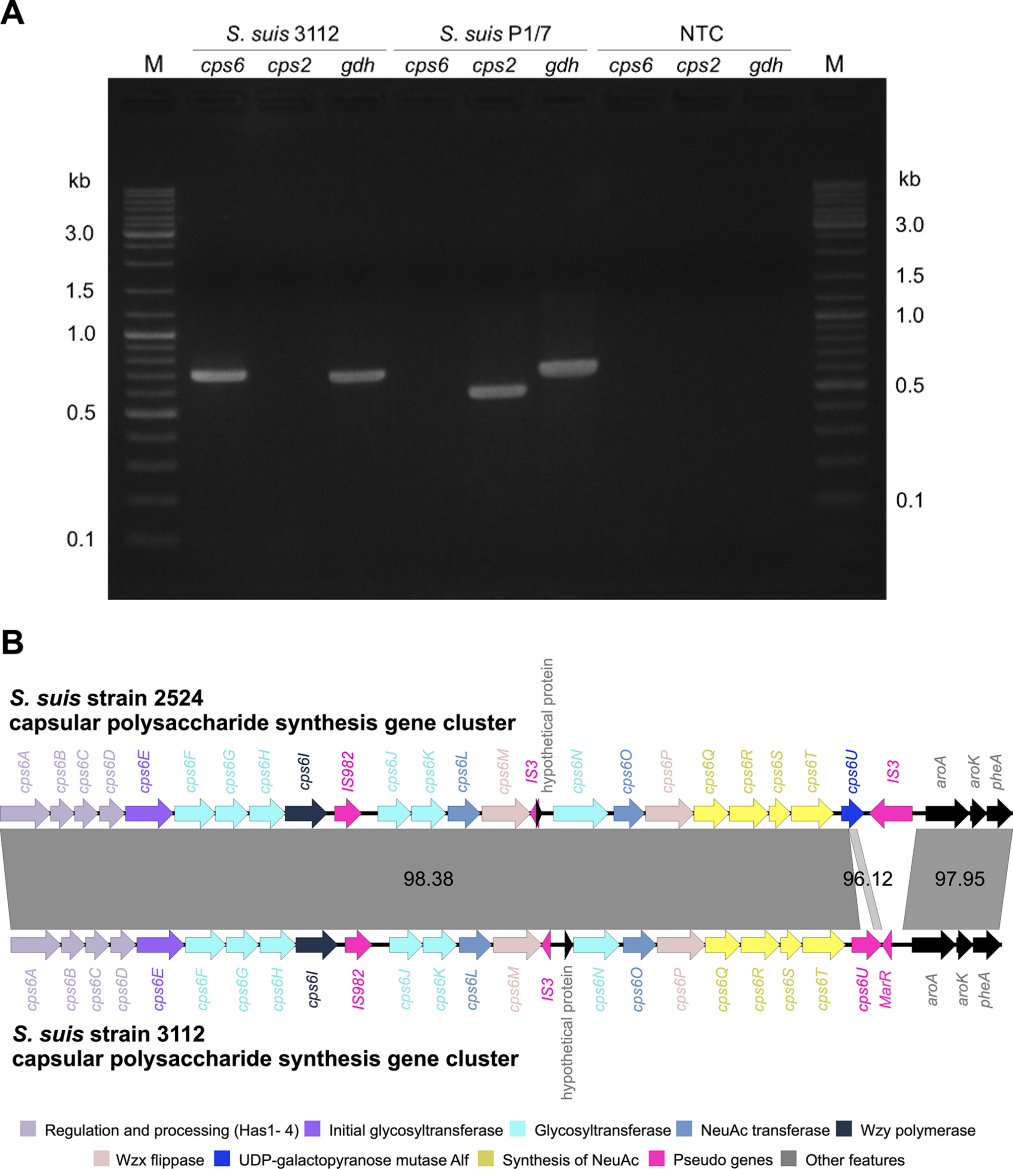
*S. suis* 3112 serotyping and its *cps* locus. **A**) PCR tests specific for *S. suis* serotype 6 (*cps6* gene; 705 bp) and serotype 2 (*cps2* gene; 577 bp). The *S. suis* serotype 2 strain P1/7 (*S. suis* P1/7) was included as an experimental control. PCR amplification of the *S. suis* glutamate dehydrogenase (*gdh*) gene (689 bp) was performed as a positive control. The results suggest that *S. suis* 3112 belongs to serotype 6, and not serotype 2. Lane M, GeneRuler 1 kb DNA Ladder (Thermo Scientific), NTC, no template control. Supplementary Fig. 1 shows a direct print-out of the gel image from the machine. **B**) Comparison of the *cps* loci between the reference *S. suis* serotype 6 strain 2524 (**top**) and *S. suis* strain 3112 (**bottom**). Genes are colour-coded based on the functions of their protein products

The synthesis of CPS involves a group of genes in the *cps* locus. The previous study reporting *S. suis* 3112 demonstrated that the bacterium does not belong to serotype 2 based on a *cps2* gene-specific polymerase chain reaction (PCR) test [10]. To serotype this bacterium, we performed a search against our bacterial genome using sequences of 33 serotype-specific PCR-primer pairs [27] with BLASTn [28]. We found that the only pair showing detectable similarity was the *cps6I* primer-pair sequences specific to serotype 6, showing 100% identity. Consistent results were obtained by PCR, using the *glutamate dehydrogenase* (*gdh*) gene as a positive *S. suis*-specific control (Fig. 2A, and Supplementary Fig. 1). Sequences of the *cps6I* and *gdh* genes in the assembled genome were validated by Sanger sequencing (Supplementary Figs. 2 and 3). The *cps* locus in the genome of *S. suis* 3112 (Fig. 1, position: 1,243,318–1,271,969) was then located by querying our genome with the *cps* locus of the reference *S. suis* serotype 6 strain 2524 (GenBank accession number: AB737818) using BLASTn [28]. A comparative analysis revealed some structural differences between the two (Fig. 2B). For instance, we found that *S. suis* 3112 has a 399-bp insertion in the middle of the *cps6U* gene as compared to the reference strain 2524, and while the reference strain 2524 has a transposase gene of the family IS3 at the 3’ end of the *cps* locus, our bacterium lacks this element, but has the *MarR* (pseudo)gene instead. Nevertheless, the overall similarity of their *cps* loci still remains high. Taken together, these results suggest that our bacterium *S. suis* 3112 belongs to serotype 6.

### *S. suis* 3112 belongs to ST 2340

Besides serotyping, another approach widely used to characterise a bacterial isolate at the subspecies level is multi-locus sequence typing (MLSTing). Under this approach, a bacterial species is divided into distinct groups called STs based on allelic profiles of several specific housekeeping genes. Currently, *S. suis* is classified into 2,705 STs using allelic profiles of *aroA*,* cpn60*,* dpr*,* gki*,* mutS*,* recA*, and *thrA* housekeeping genes (PubMLST, Jolley et al. [29]; access date: 29/02/2024), originally developed by King et al. [30]. Compared with serotyping, MLSTing provides a higher resolution classification scheme, enabling more detailed classification and characterisation of bacterial isolates. For instance, while both ST1 and ST7 belong to serotype 2, it has been reported that the former is globally distributed, while the latter is predominantly found only in China [1], providing important insights into the epidemiological distribution of the bacteria with higher resolution.

To determine the ST of *S. suis* 3112, its assembled genome was analysed using the online database PubMLST [29]. Our analysis revealed that *S. suis* 3112 belongs to ST 2340, and the alleles of the genes *aroA*,* cpn60*,* dpr*,* gki*,* mutS*,* recA*, and *thrA* of *S. suis* 3112 were determined as 490, 646, 30, 559, 546, 394, and, 428, respectively. The sequences of these genes validated by Sanger sequencing (Supplementary Figs. 4–10), thereby confirming the typing result.

### Phylogenetic analysis of *S. Suis* 3112

Phylogenetic analysis was performed to determine a probable origin of *S. suis* 3112. 90 whole genome sequences of *S. suis* compiled by Dong et al. [31] were randomly selected, and used as contextualising genomes, comprising 30 genomes of human isolates, 30 genomes of diseased-pig isolates, and 30 genomes of healthy-pig isolates (Supplementary Table 3), as well as the genome of the serotype-6 reference strain 2524 isolated from a diseased pig. All publicly available whole genome sequences of *S. suis* samples obtained from non-pig/non-human animals on the NCBI Assembly database were also included in this analysis, including the genomes of 1 cattle isolate, 1 river otter isolate, 1 lamb isolate, 1 cat isolate, and 4 red junglefowl isolates (Supplementary Table 3). The analysis was conducted using an alignment of variant sites sampled across the entire genome, excluding sites with > 25% missing bases, constructed by using *S. suis* 3112 as the reference genome anchor. By comparing to *S. suis* 3112, 16 samples were found to have an overall *S. suis* 3112 coverage < 75%, and thus were excluded from further analysis (Supplementary Table 3). The remaining contextualising genomes were 29 human samples, 29 diseased-pig samples, 22 healthy-pig samples, 1 cat sample, 1 otter sample, and 1 red junglefowl sample. While this contextualising dataset might not cover the entire diversity of *S. suis*, it should be sufficient for determining a probable origin of *S. suis* 3112 as it contained some of the most similar genomes to that of our bacterium already. Significant evidence for recombination (p-value < 0.00001) was detected in the alignment (Supplementary Data 1) using the PHI test [32]. Thus, we opted for phylogenetic network analysis as the method of choice for this study.

**Fig. 3:**
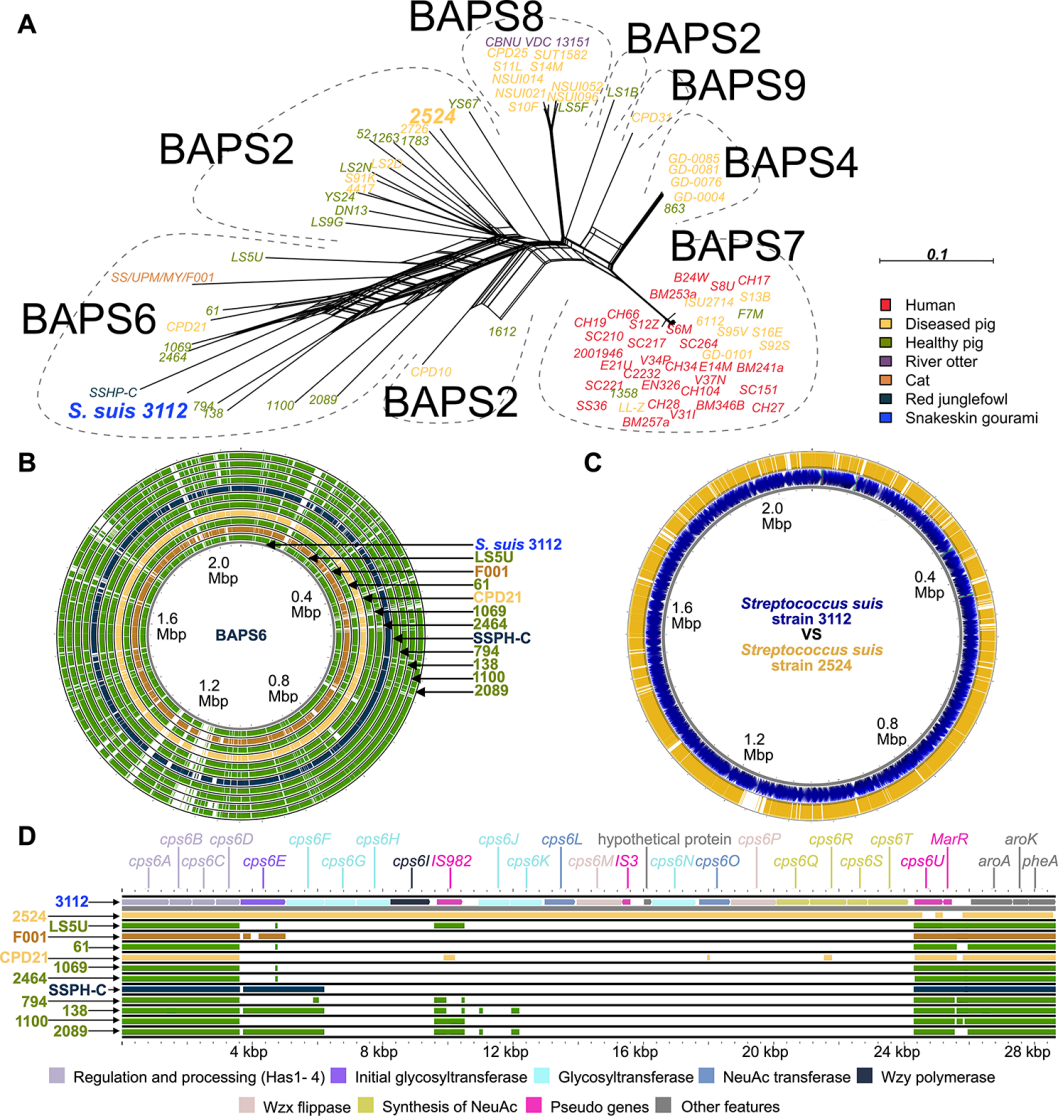
Phylogenetic network analysis, whole genome comparison, and *cps* locus comparison of *S. suis* 3112 with closely related isolates. **A**) Phylogenetic network analysis included 29 human samples (red), 28 diseased-pig samples (yellow), 22 healthy-pig samples (green), 1 otter sample (purple), 1 cat sample (orange), and 1 red junglefowl sample (dark navy) as contextualising genomes (Supplementary Table 3). The serotype-6 reference strain, *S. suis* 2524 (indicated with a larger font size), was also included in this analysis. Our bacterial isolate *S. suis* 3112 is highlighted in blue (host = Snakeskin gourami). The network was estimated from an alignment of variant sites sampled from the entire bacterial genome constructed by using our *S. suis* 3112 sample as the reference anchor genome, excluding sites with > 25% missing bases (Supplementary Data 1). The Neighbour-net method implemented in SplitsTree4 [33] was used to reconstruct the network, and the GTR + F + Γ nucleotide substitution model was used to best approximate the best-fit nucleotide substitution model, TVM + F + ASC + R6, identified under the Bayesian information criterion by ModelFinder [34] implemented in IQ-TREE2 [35] (see Methods). Bacterial groups identified by the Bayesian analysis of population structure (BAPS) by Dong et al. [31] are indicated. The scale bar represents 0.1 substitutions per site. See Supplementary Data 2 for the raw NEXUS network file. **B**) Whole genome comparison of *S. suis* 3112 against BAPS6 samples. **C**) Whole genome comparison of *S. suis* 3112 and 2524. In both analyses, *S. suis* 3112 was used as the reference, and colours of the genomes indicate the bacterial hosts (see key in **A**). **D**) Comparison of the *cps* loci of *S. suis* 3112 (1st row), *S. suis* 2524 (2nd row), and BAPS6 samples (3rd to 13th rows). The *cps* locus of *S. suis* 3112 served as the anchor reference in the pairwise sequence similarity detection (e-value threshold = 0.1). Genes are colour-coded based on the functions of their protein products. Colours of the genomes indicate the bacterial hosts (see key in **A**)

Dong et al. [31] classified *S. suis* into 9 sub-populations based on the similarity among their single nucleotide polymorphisms using a Bayesian analysis of population structure (BAPS), named BAPS1 to 9. According to this classification scheme, we found that *S. suis* 3112 fell within the diversity of BAPS6 members (Fig. 3), predominantly consisting of healthy-pig bacterial isolates, as well as the two cat and red junglefowl isolates. This phylogenetic pattern suggests an ultimate swine (as opposed to human) origin of *S. suis* 3112. This finding aligns with the information that the bacterial isolate was obtained from a fish farm where the fish rearing water was likely contaminated with pig excrements, corroborating the hypothesis.

Further examination of the network revealed extensive reticulation branches connecting *S. suis* 3112 and other BAPS6 members, suggesting potential both deep and recent genetic exchanges among them. Interestingly, although *S. suis* 3112 and BAPS6 isolates exhibited high detectable similarity across their entire genomes (Fig. 3B; range of average nucleotide identities (ANIs): 95.45–96.06%; Supplementary Table 4), their *cps* loci showed very little nucleotide-level similarity among them (Fig. 3D). Out of the 11 contextualising BAPS6 isolates in our dataset, we were able to serotype 6 of them by querying their assembled genomes against serotype-specific PCR-primer sequences proposed by Liu et al. [27] and Kerdsin et al. [36] using BLASTn [28]. We found that, indeed, none of them belonged to serotype 6, but instead were found to belong to at least 3 distinct serotypes (serotypes 5: isolate 2464; serotype 15: isolates 138, and 2089; serotype 31: isolates 61, 1069, and LS5U; unidentifiable: isolates 794, 1100, CPD21, SS/UPM/MY/F001, and SSHP-C; Supplementary Table 5). On the other hand, while the *cps* loci of *S. suis* 3112 and 2524 were highly similar (Fig. 2, and 3D), they did not cluster together on the phylogenetic network (although a high degree of genome-wide similarity comparable to those against BAPS6 isolates could still be observed, Fig. 3C; ANI: 95.40%, Supplementary Table 4). Altogether, these findings support the notion of extensive horizontal gene transfer, involving the *cps* locus. Indeed, *cps* gene recombination has been reported in some *S. suis* serotypes, and is recognised as a mechanism generating diversity of *cps* loci in *S. suis* [37]. A high recombination rate has been suggested to promote host generalism and expansion of the host range in bacteria [38]. The observation that *S. suis* 3112, which must have undergone a (series of) inter-species transmission(s) in the past, belongs to a group of *S. suis* with a pronounced level of recombination as well as containing two other non-pig/non-human samples is therefore a noteworthy one. However, the exact role of recombination in this process (if any) remains unclear, and further investigation is required to shed more light on the specific mechanisms underlying these genetic exchanges and host jump.

### Antimicrobial drug susceptibility testing

**Fig. 4:**
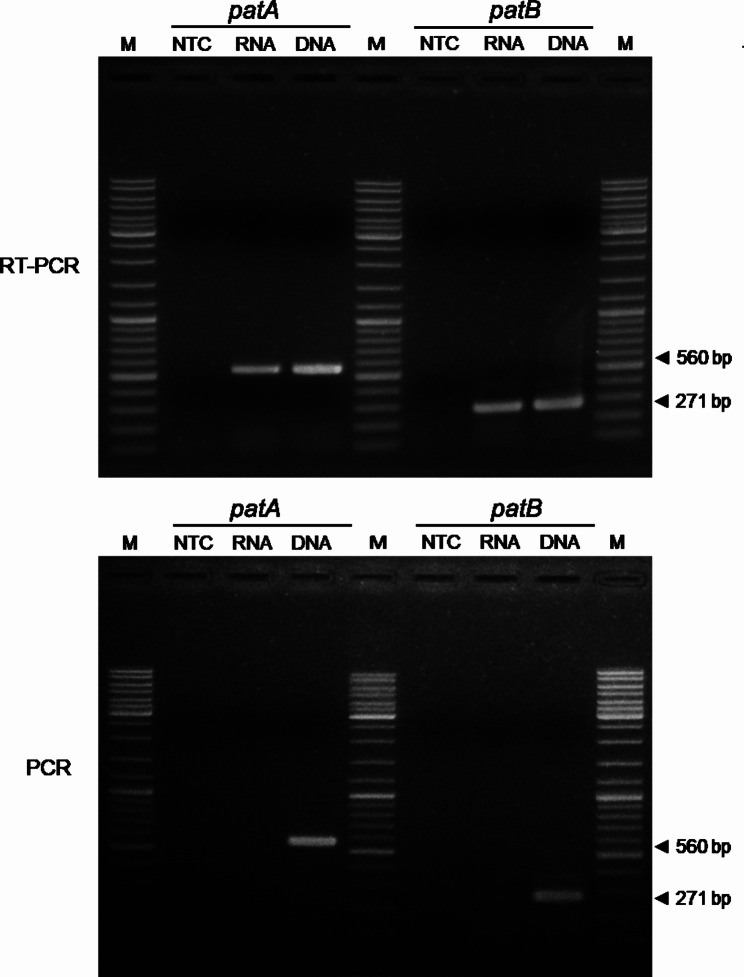
Expression of *patA* and *patB* transcripts in *S. suis* 3112. The presence of *patA* and *patB* transcripts in *S. suis* 3112 RNA was evaluated using RT-PCR (**top**). No amplicons were obtained from PCR reactions without reverse transcription (**below**), confirming the absence of DNA contamination in the RT-PCR reactions. *S. suis* 3112 DNA was used as an amplification control. M, DNA marker; NTC, no template control. Supplementary Fig. 13 shows a direct print-out of the gel image from the machine

The resistance of *S. suis* to antimicrobial drugs is a global issue, most commonly against tetracyclines and macrolides, but also to aminoglycosides, fluoroquinolones, amphenicols, and glycopeptides [39, 40]. Our analysis identified two potential drug resistance genes in the genome of *S. suis* 3112 (Fig. 1, right), namely *patA* (position: 1,913,151–1,914,857), and *patB* (position: 1,911,366–1,913,150). These genes encode ATP-binding cassette efflux pumps, PatA and PatB, which are known to confer resistance to fluoroquinolone antibiotics, particularly norfloxacin, followed by ciprofloxacin, gemifloxacin, levofloxacin, and moxifloxacin [14–16]. The average short-read assembly depth for *patA* and *patB* was 468× and 456×, respectively, strongly supporting their presence within the bacterial genome, and the gene sequences could be validated by Sanger sequencing (Supplementary Figs. 11 and 12). Reverse transcription (RT)-PCR analysis revealed the expression of these two genes in our bacterium (Fig. 4, and Supplementary Fig. 13).

**Fig. 5:**
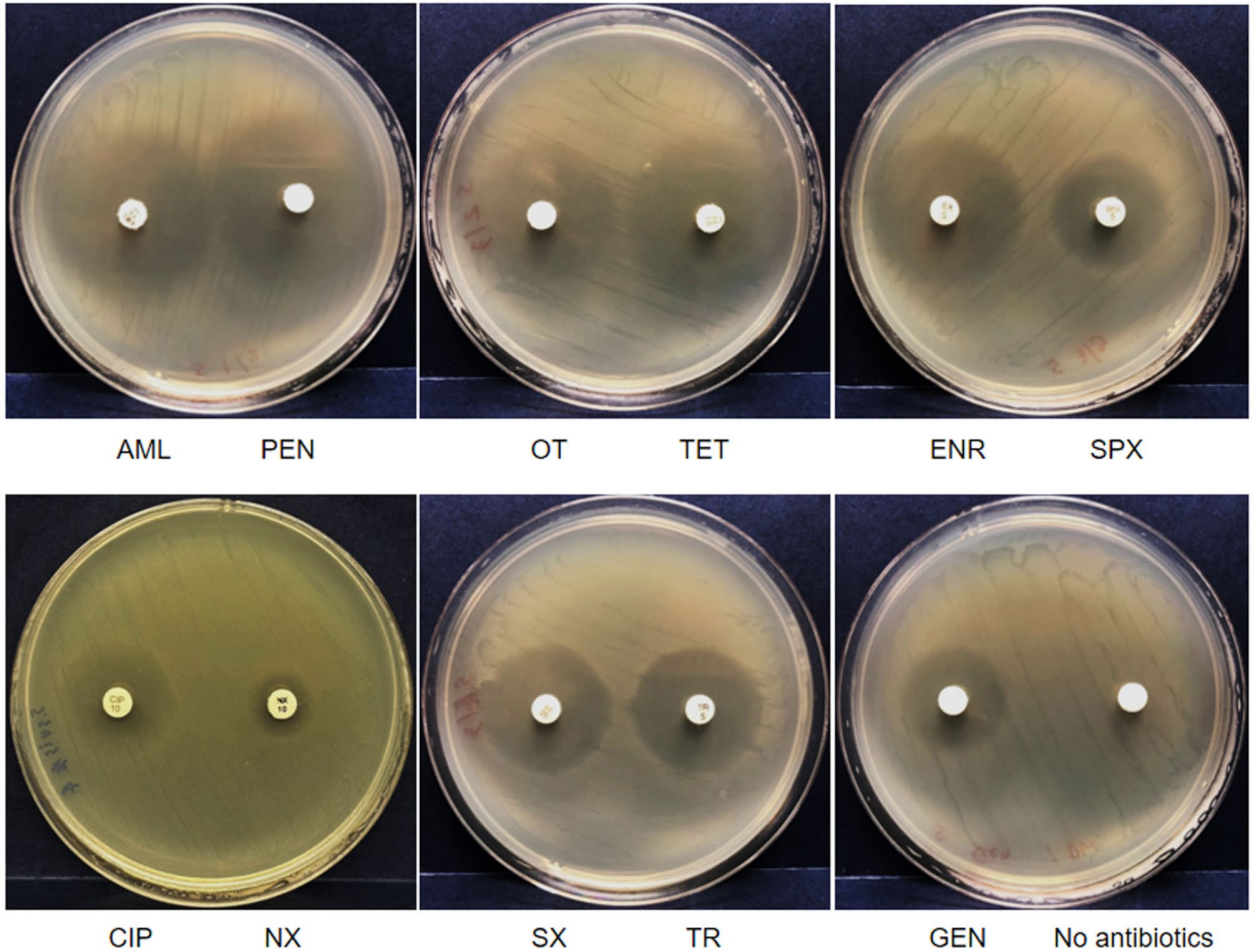
One replicate of disk diffusion assays assessing the susceptibility of *S. suis* 3112 to eleven antimicrobial drugs from five distinct classes. The tested drugs included β-lactams (AML: amoxicillin, and PEN: penicillin), tetracyclines (OT: oxytetracycline, and TET: tetracycline), fluoroquinolones (ENR: enrofloxacin, SPX: sparfloxacin, CIP: ciprofloxacin, and NX: norfloxacin), sulphonamides (SX: sulphamethoxazole, and TR: trimethoprim), and an aminoglycoside (GEN: gentamicin). The results show that *S. suis* 3112 is resistant to NX, but susceptible to all other drugs tested. See Table 3 for detailed antimicrobial susceptibility testing results. *Staphylococcus aureus* 1020 was used as an experimental control (Supplementary Fig. 14, and Supplementary Table 6)

Antimicrobial susceptibility testing was conducted on *S. suis* 3112 against five classes of drugs, including β-lactam (AML: amoxicillin, and PEN: penicillin), tetracycline (OT: oxytetracycline, and TET: tetracycline), fluoroquinolone (ENR: enrofloxacin, CIP: ciprofloxacin, NX: norfloxacin, and SPX: sparfloxacin), sulphonamide (SX: sulphamethoxazole, and TR: trimethoprim), and aminoglycoside (GEN: gentamicin) (Table 3; Fig. 5). *Staphylococcus aureus* 1020 was used as an experimental control (Supplementary Fig. 14, and Supplementary Table6). These drugs are permitted, and are commonly used in aquaculture (AML, OT, ENR, and TR), pig farming (AML, PEN, OT, TET, ENR, and GEN), and the treatment of human diseases (AML, PEN, TET, CIP, NX, SX, TR, and GEN) [41–43].

**Table 3:** Antimicrobial susceptibility testing results for *S. Suis* 3112

Antimicrobial class	Antimicrobial agent	Amount of the drug in the disk	Inhibition zone diameter (mm)*				Observed inhibition zone diameter (mm)	Susceptibility*
			S	I		*R*		
β-lactam	Amoxicillin (AML)	25 µg	*≥* 26		19–25	*≤* 18	36.03 *±* 1.42	S
	Penicillin (PEN)	10 Units	*≥* 28		22–27	*≤* 21	37.53 *±* 1.45	S
Tetracycline	Oxytetracycline (OT)	30 µg	*≥* 29		19–28	*≤* 18	34.30 *±* 0.70	S
	Tetracyclin (TET)	30 µg	*≥* 23		19–22	*≤* 18	30.00 *±* 0.61	S
Fluoroquinolone	Enrofloxacin (ENR)	5 µg	*≥* 23		17–22	*≤* 16	34.76 *±* 0.64	S
	Sparfloxacin (SPX)	5 µg	*≥* 19		16–18	*≤* 15	25.90 *±* 0.10	S
	Ciprofloxacin (CIP)	10 µg	≥ 21		16–20	*≤* 15	22.83 *±* 0.31	S
	Norfloxacin (NX)	10 µg	≥ 17		13–16	*≤* 12	10.83 *±* 0.55	R
Sulfonamides	Sulphamethoxazole (SX)	25 µg	*≥* 25		21–24	*≤* 20	30.10 *±* 0.30	S
	Trimethoprim (TR)	5 µg	*≥* 16		11–15	*≤* 10	27.93 *±* 0.42	S
Aminoglycoside	Gentamicin (GEN)	10 µg	*≥* 16		13–15	*≤* 12	21.76 *±* 0.40	S

*S: Sensitive; I: Intermediate; R: Resistant

Disk diffusion assays were conducted to evaluate the susceptibility of *S. suis* 3112 to eleven antimicrobial drugs from five distinct classes. The experiment was performed in triplicate, and the mean diameters of the inhibition zones (mm) are reported with standard deviations. Zone diameter interpretive standard chart is provided in the table. Images from one replicate of the disk diffusion assays are shown in Fig. 5. *Staphylococcus aureus* 1020 was used as an experimental control (Supplementary Fig. 14, and Supplementary Table 6)

The results demonstrate that *S. suis* 3112 is resistant only to norfloxacin, but still susceptible to all other drugs tested, including the other three fluoroquinolones (ciprofloxacin, enrofloxacin, and sparfloxacin). Indeed, it has been observed that resistance mediated by PatA/PatB does not affect all fluoroquinolones equally, with the most hydrophobic molecules being the least affected ones [14]. Enrofloxacin and sparfloxacin are relatively more hydrophobic compared to, for example, ciprofloxacin and ofloxacin [44, 45], which have been classified as molecules of intermediate lipophilicity, while norfloxacin is a hydrophilic derivative [46], explaining our observations.

## Conclusions

This study presents a whole genome analysis of *S. suis* 3112, a novel strain of *S. suis* recently identified as the causative agent of lethal streptococcosis in snakeskin gourami, an economically important fish species in Southeast Asia [10]. Our analysis indicated that *S. suis* 3112 belongs to serotype 6, and ST 2340. Results from phylogenetic analysis suggest that the bacterium belongs to a group of bacteria with a considerably high level of recombination containing two other non-pig/non-human isolates, and likely originated from cross-transmission ultimately from pigs (as opposed to humans) to fish. Within its genome, we identified two drug resistance genes, namely *patA* and *patB*, which are known to confer resistance to fluoroquinolone antibiotics. We confirmed the expression of these genes in our bacterium, and found that *S. suis* 3112 is resistant to norfloxacin, while still remains sensitive to the other three fluoroquinolones tested, including ciprofloxacin, enrofloxacin, and sparfloxacin, as well as four other classes of antimicrobial drugs, including β-lactam, tetracycline, sulphonamide, and aminoglycoside.

In summary, our results improve our understanding of this newly identified strain of *S. suis* in fish, and the presence of drug resistance genes underscores the need for continuous vigilance of this bacterium. Further research on its epidemiology, evolution, host-interactions, and adaptability, is warranted to better understand the mechanisms of cross-species transmission, and to assess its prevalence, distribution, and potential impacts on fish populations and farming. Discovery of additional samples through these studies will aid in establishing effective disease treatment and management strategies against this new fish pathogen.

## Materials and methods

### *S. suis* 3112 DNA extraction

DNA extraction of *S. suis* 3112 was performed using a modified Gram-positive bacteria extraction protocol described by Salvà-Serra et al. [47]. A culture of *S. suis* 3112 was prepared by inoculating a single bacterial colony into 5 mL of tryptic soy broth (TSB, Difco, BBL) medium. The culture was incubated by shaking at 250 rpm overnight at 30 °C. After centrifugation at 5,000× g for 5 min at 4 °C, the pellet was resuspended in 300 µL of EDTA-saline (0.01 M EDTA and 0.15 M NaCl), 50 µL of 110 mg/mL lysozyme, and 10 µL of 20 mg/mL RNase A. The mixture was incubated at 37 °C for 2 h. Next, the mixture was mixed with lysis buffer (160 µL of 20% SDS, 20 µL of 5 mg/mL proteinase K) and incubated at 65 °C for 30 min. After incubation, the mixture was added to 0.5 volume of 5 M NaCl, vortexed for a few seconds, and then subjected to 1 volume of phenol: chloroform: isoamyl alcohol (25:24:1) followed by centrifugation at 10,000× g for 15 min. The upper phase was transferred to a new tube, and this step was repeated twice, with the second time using chloroform: isoamyl alcohol (24:1). DNA was precipitated from the upper phase using 0.1 volume of 3 M sodium acetate (pH 5.2), and 2 volumes of cold absolute ethanol, and then incubated overnight at -20 °C. The DNA pellet was washed with cold 70% ethanol, dried, and dissolved in DNase-free water. The quality and quantity of DNA were measured using the Qubit Fluorometer, and the DNA samples were stored at -20 °C.

### 2nd generation sequencing

Genomic DNA of *S. suis* 3112 was sent to Novogene through Ward Medic Ltd. Part, Thailand for sequencing. Briefly, the extracted genomic DNA of *S. suis* 3112 was randomly sheared into short fragments. The obtained fragments were then end-repaired, A-tailed, and further ligated with Illumina adapters. The fragments with adapters were subsequently amplified by using PCR, size selected, and purified before being subjected to Illumina sequencing.

### 3rd generation sequencing

Sequencing library preparation was carried out using the Nanopore Genomic Sequencing Kit SQK-LSK109 and native barcoding expansion 1–12 (EXP-NBD104) protocols from Oxford Nanopore Technologies. The NEBNext FFPE DNA Repair and NEBNext Ultra II End Repair/dA Tailing kit (New England Biolabs, Ipswich, USA) were used to prepare 1 µg of sheared *S. suis* 3112 genomic DNA (1 µg DNA in 48 µL of nuclease-free water, 3.5 µL of NEBNext FFPE DNA Repair buffer, 3.5 µL of Ultra II End-Prep reaction buffer, 3 µL of Ultra II End-Prep Enzyme Mix, and 2 µL of NEBNext FFPE DNA repair Mix, in a total volume of 60 µL). The reaction mixture was incubated at 20 °C for 5 min and heat-inactivated at 65 °C for 5 min. The DNA was purified using a 1:1 volume of Agencourt AMPure XP beads (A63880, Beckman Coulter) according to the manufacturer’s instructions and eluted in 25 µL of nuclease-free water.

To ligate native barcode adapters to the shared DNA, 22.5 µL of 500 ng of end-prepared DNA were mixed with 2.5 µL of Native Barcode, and 25 µL of Blunt/TA Ligase Master Mix in a total volume of 50 µL. The mixture was incubated at room temperature for 10 min, purified using a 1:1 volume of AMPure XP beads, and then eluted in 26 µL of nuclease-free water. 350 ng of the barcoded DNA sample were then mixed with 350 ng of another barcoded DNA sample (from another study), to prepare a total of 700 ng in 65 µL of pooled barcoded DNA sample.

The sequencing adaptors were then ligated to the pooled barcoded DNA sample by adding 5 µL of Adapter Mix II (AMII), 20 µL of NEBNext Quick Ligation reaction buffer (5×), and 10 µL of Quick T4 DNA Ligation (New England Biolabs, Ipswich, USA) to the barcoded DNA sample. The mixture was incubated at room temperature for 10 min. The DNA library was further purified using AMPure XP beads in a 1:1 ratio of beads to sample volume, and eluted in 15 µL of nuclease-free water. DNA concentrations at each step were measured using the Qubit Fluorometer.

To sequence the bacterial genome, a total DNA sequencing library of 12 µL was mixed with 37.5 µL of the sequencing buffer and 25 µL of loading beads (SQK-MAP006, ONT), and immediately loaded onto a SpotON Flow cell R9.4.1 on a MinION MK1B controlled by MinKNOW version 21.02.1 for sequencing. Data acquisition and base-calling were performed using Guppy v4.3.4 + ecb28058b with the *Basecall_Barcoding* workflow.

### *De novo* whole genome assembly

The quality of the short reads was assessed using FastQC [48], and Trimmomatic [49] was employed to clean the reads with the setting “*SLIDINGWINDOW:4:30 MINLEN:70*”. For the long reads, PoreChop [50] was used to check and remove remaining sequencing adapters, and CANU’s [51] *corrected* function was applied to correct the reads. *De novo* genome assembly was performed using Unicycler V.0.4.8 [52] on the clean long reads with the “*bold*” mode and an estimated genome assembly size of 2.1 Mbp.

### Genome annotation

Genome annotation was conducted using Prokaryotic Genome Annotation Pipeline v6.1 [53]. Potential drug resistance genes were identified by using CARD RGI v5.2.1 [13], available from Proksee (https://proksee.ca). Virulence factors, and mobile genetic elements were detected using VFanalyzer [11], and mobileOG-db V1.1.2 [12], respectively.

### Serotyping

Serotyping was performed by querying the assembled genome against a set of 33 serotype-specific PCR-primer pair sequences [27] using BLASTn [28]. A detectable similarity to our genome was found only for the pair corresponding to serotype 6. PCR tests were conducted to confirm the result.

In the PCR-based serotyping, two primer pairs specific to serotypes 2 and 6 were used (Table 4). Each 25 µL PCR reaction contained 1x AccuStartTM II GelTrack PCR SuperMix (Catalogue number: Quantabio 95136-100), 200 nM of each of the forward and reverse primers, 200 ng of DNA template, and distilled water to adjust the final volume. The PCR cycling conditions consisted of one initial round of denaturation of DNA templates and primers at 94 °C for 5 min, followed by 35 PCR amplification cycles at 94 °C for 30 s, 58 °C for 30 s, 72 °C for 30 s, with a final extension at 72 °C for 5 min. PCR products were electrophoresed on a 1% (w/v) agarose gel, stained with ethidium bromide, and visualised under UV light. The experiment was also performed using *S. suis* serotype 2 strain P1/7 for comparison. Amplification of the *S. suis* glutamate dehydrogenase (*gdh*) gene [54] was performed as a control. The picture of the gel was captured using the Gel Documentation Systems (Aplegen, USA) machine.

### Characterisation of the *capsular polysaccharide* (*cps*) locus

To locate the *cps* locus, we searched in our genome for a genomic region showing high similarity to the reference *cps* locus of *S. suis* serotype 6 strain 2524 (GenBank accession number: AB737818) using BLASTn [28]. The identified locus was compared against the reference locus using Easyfig v2.2.5 [55], and the genes in the reference *cps* locus of the strain 2524 were reannotated using Prokaryotic Genome Annotation Pipeline v6.1 [53] for the comparison. Additionally, we compared the *cps* locus of our bacterial isolate with those of several closely related healthy-pig *S. suis* isolates using the same programme.

### Multi-locus sequence typing

The assembled genome sequence was analysed to determine the sequence type of *S. suis* 3112 using the online database PubMLST [29]. The typing was based on the allelic profile of seven housekeeping genes, including *aroA*, *cpn60*, *dpr*, *gki*, *mutS*, *recA*, and *thrA*.

### Phylogenetic analysis

90 whole genome sequences of *S. suis* were randomly selected from the list compiled by Dong et al. [31], comprising 30 genomes of human isolates, 30 genomes of diseased-pig isolates, and 30 genomes of healthy-pig isolates. In addition, the genome of the reference serotype 6 *S. suis* strain 2524 (from a diseased pig), and all publicly available whole genome sequences of *S. suis* collected from non-pig/non-human animals were downloaded from the NCBI Assembly database (access date: 03/02/2024), including those of 1 cattle isolate, 1 river otter isolate, 1 lamb isolate, 1 cat isolate, and 4 red junglefowl isolates. All of these sequences were then compared to *S. suis* 3112 to perform variant calling, using *snippy-multi* implemented in *snippy* [56]. 16 samples were found to have an overall coverage against the *S. suis* 3112 genome < 75%, and thus were removed from further analysis. *snippy-core* in *snippy* [56] was then used to construct a whole-genome alignment, and the alignment was further processed by *snp-sites* to generate an alignment containing only variant sites. Finally, *clipkit* [57] with the ‘gappy mode’ was used to remove positions with > 25% missing bases. The final alignment contained 84 sequences, and 187,731 SNP positions. The PHI test [32] in RDP5 [58] was employed to detect potential recombination within the alignment, and significant evidence was found (p-value < 0.00001). Thus, phylogenetic network analysis was chosen as the method of choice for this study.

Phylogenetic network reconstruction was performed using SpiltsTree4 [33] with the Neighbour-net method. ModelFinder [34] in IQ-TREE2 [35] determined TVM + F + ASC + R6 as the best-fit nucleotide substitution model for our alignment under the Bayesian information criterion (maximum likelihood relative rate estimates: A<-> C, 1.00750; A<-> G, 5.61025; A<-> T, 1.56623; C<-> G, 0.23968; C<-> T, 5.61025; and G<-> T: 1.0000; nucleotide frequency: A, 0.240; C, 0.258; G, 0.266; T, 0.237; and site-wise rate variation distribution (proportion, rate): (0.637, 0.242), (0.227, 1.449), (0.122, 3.468), (0.011, 3.621), (0.001, 9.940), (0.002, 22.013)). In the phylogenetic network reconstruction, we therefore used the GTR + F + Γ(α = 0.9058612) model with the rate parameter values described above to best approximate the best-fit nucleotide substitution model.

### Antimicrobial susceptibility tests

A single colony of *S. suis* 3112 was cultured overnight in 5 mL TSB at 30 °C with shaking at 250 rpm. Then, 1 mL of the overnight culture was harvested, inoculated into 100 mL of prewarmed TSB, and incubated under the same conditions for 6 h. The bacterial cell suspension was adjusted to have an OD_600_ of 2, corresponding to approximately 2.5 × 10^8^ cfu/mL. Subsequently, the cell suspension was harvested and spread onto Mueller Hinton agar plates (Difco, BBL). Disks containing antimicrobial agents, including amoxicillin (AML, 25 µg), penicillin (PEN, 10 Units), oxytetracycline (OT, 30 µg), tetracycline (TET, 30 µg), enrofloxacin (ENR, 5 µg), sparfloxacin (SPX, 5 µg), ciprofloxacin (CIP, 10 µg), norfloxacin (NX, 10 µg), sulphamethoxazole (SX, 25 µg), trimethoprim (TR, 5 µg), and gentamicin (GEN, 10 µg), were placed on the bacterial agar plate using a disk dispenser (HiMedia). The plates were then incubated at 30 °C for 18 h. The diameter of the inhibition zone of *S. suis* 3112 was measured and interpreted as susceptible, intermediate, or resistant based on established interpretive criteria for *S. suis* or other *Streptococcus* spp. as specified by the Clinical and Laboratory Standards Institute (VET08-ED4 and M100-ED28). The experiment was conducted in triplicate, and *Staphylococcus aureus* 1020 was used as the control.

### Detection of *patA* and *patB* expression in *S. Suis* 3112

Total RNA was extracted from a 5-mL overnight culture of *S. suis* 3112 using TRIzol Reagent (Invitrogen) following the manufacturer’s instructions. To remove any potential contaminating DNA, the reaction mixture containing 1 µg of the total extracted RNA, 10 units of DNase I, and 1× DNase I reaction buffer (Thermo Fisher Scientific) was incubated at 37 °C for 30 min. The DNA-free RNA was then purified again using TRIzol Reagent. The presence of *patA* and *patB* transcripts in *S. suis* 3112 was examined using reverse transcription (RT)-PCR. The RT-PCR reactions consisted of 200 ng of RNA, 400 nM of each respective *pat* primer pair (Table 4), 0.5 µL SuperScript™ III RT/Platinum™ Taq Mix (Invitrogen), and 1× reaction buffer. The amplification protocol involved reverse transcription at 50 °C for 10 min, RT inactivation at 94 °C for 2 min, followed by 35 cycles of denaturation at 94 °C for 30 s, annealing at 58 °C for 30 s, extension at 72 °C for 30 s, and a final extension step at 72 °C for 2 min. As a control, DNA from *S. suis* 3112 obtained from the aforementioned step was also subjected to the RT-PCR analysis. To confirm the absence of DNA contamination, the extracted *S. suis* 3112 RNA was subjected to PCR reactions as described in the serotyping method mentioned earlier, except that the *patA* or *patB* primers were used. The RT-PCR and PCR products were separated by electrophoresis on a 1% (w/v) agarose gel, stained with ethidium bromide, and visualised under UV light. The pictures of the gels were captured using the Gel Documentation Systems (Aplegen, USA) machine.

### Validation of gene sequences in the assembled genome using Sanger sequencing

Sanger sequencing was used to validate sequences of the key genes mentioned in the study, including the serotype-6 specific gene *cps6*, the general *S. suis* species specific gene *gdh*, the seven housekeeping genes defining the bacterial ST (*aroA*, *cpn60*, *dpr*, *gki*, *mutS*, *recA*, and *thrA*), and the two detected drug resistance genes *patA*, and *patB*. See Table 4 for the primers used.

**Table 4:** Primers used in this study

Gene	Primer name	Primer positions (referring to CP097577.2)*	Primer sequence (5’–3’)	Product size (bp)	Reference
*cps2J*	cps2J-F	N/A	TGATAGTGATTTGTCGGGAGGG	577	[59]
	cps2J-2	N/A	GAGTATCTAAAGAATGCCTATTG		
*cps6I*	cps6I_F	1,263,552–1,263,571	TGGTGTCTTTCTACCTGCAA	705	[27]
	cps6I_R	1,262,867–1,262,886	TCACCAAGATACGTGAACCA		
*gdh*	JP4	232,449–232,468	GCAGCGTATTCTGTCAAACG	689	[54]
	JP5	233,118–233,137	CCATGGACAGATAAAGATGG		
*aroA*	aroA_571-F	1,244,938–1,244,957	TTCCATGTGCTTGAGTCGCT	571	This study
	aroA_571-R	1,245,489–1,245,508	TCTGGACAAACTGAACGCGA		
*cpn60*	cpn60_513-F	133,994–134,013	CGGTACAACAACTGCCACTG	513	
	cpn60_513-R	134,487–134,506	AGAGCTTCGCCATCCACATC		
*dpr*	dpr_511-F	1,546,812–1,546,835	TGGACCCTTCATCTATTTACAACT	511	
	dpr_511-R	1,547,303–1,547,322	TCTCCAGCAGAAATTGCGTC		
*gki*	gki_598-F	981,875–981,894	CCCAGAACGATGTAGGCAGG	598	
	gki_598-R	982,453–982,472	TTGGTATGGGGTCTCCAGGT		
*mutS*	mutS_573-F	2,003,137–2,003,156	TCCGCAATAGTCGCATACCC	573	
	mutS_573-R	2,003,690–2,003,709	TCAAGCGGGAAGTAGTGCAG		
*recA*	recA_640-F	67,672–67,691	GACTCTGGTGCGGTTGACTT	640	
	recA_ 40-R	68,292–68,311	CTTCTTCGGTCGCAACCTCT		
*thrA*	thrA_586-F	1,645,248–1,645,267	TGCACCTGGAAAACGCAATG	586	
	thrA_586-R	1,645,814–1,645,833	GATACCAGGATGGGCAGCAA		
*patA*	patA-F	1,913,292–1,913,310	CTTCTGAGCAACGATGACC	560	
	patA-R	1,913,833–1,913,851	ATCCGCATGATACTGAGCC		
*patB*	patB-F	1,911,855–1,911,874	ACCGACCTTATCCCGCAAAC	271	
	patB-R	1,912,105–1,912,125	GCAAAACGCTCCGCTATTTAC		

*N/A, not applicable

The gene fragments were first amplified by PCR. Each 50 µL PCR reaction contained 200 ng of *S. suis* 3112 DNA, 300 nM of the gene’s forward and reverse primer each, and 1× KOD OneTM PCR Master Mix containing a proofreading DNA polymerase (Toyobo, Japan, Catalog number: KMM-101). A reaction without a DNA template was used as the PCR negative control. The PCR amplification was carried out for 40 cycles of denaturation at 98 °C for 10 s, annealing at 58 °C for 5 s, and extension at 68 °C for 10 s. The amplified PCR products were separated by using a 1% (w/v) agarose gel, stained with ethidium bromide, and visualised under UV light. Expected DNA bands were extracted using the NucleoSpin^®^ Gel and PCR Clean-up (Takara, USA, Catalog number: 740609.50) according to the manufacturer’s instructions. After quantification by Nanodrop spectrophotometry (Thermo Scientific, USA), the purified PCR products were bi-directionally sequenced using Sanger sequencing (Macrogen, South Korea). The obtained sequencing data were inspected in Geneious Prime (Biomatters, USA), and the consensus sequences were compared to our *S. suis* 3112 genome (CP097577.2). The results showed a 100% identity match for all eleven sequences (Supplementary Table 7), validating the gene sequences within the assembled genome.

### Electronic supplementary material

Below is the link to the electronic supplementary material.

Supplementary Material 1

Supplementary Material 2

Supplementary Material 3

Supplementary Material 4

## Data Availability

Short-read and long-read datasets generated during this study were deposited in the National Center for Biotechnology Information (NCBI)’s Sequence Read Archive database under the BioProject PRJNA838757, accession numbers SRR24927969 and SRR24941354, respectively. The complete genome of *S. suis* 3112 (BioSample: SAMN28462640) has been submitted to the NCBI GenBank database under the accession number CP097577.2. Data generated or analysed during this study are included in this published article and its supplementary information files.
