# Supplementary material for: Whole genome sequence and characterisation of Streptococcus suis 3112, isolated from snakeskin gourami, Trichopodus pectoralis
Source: BMC Genomics. 2024 Aug 28;25:808. doi: 10.1186/s12864-024-10736-x (PMC11351508; doi:10.1186/s12864-024-10736-x)
Supplement: Supplementary file 1 — Supplementary Material 1 [file 12864_2024_10736_MOESM1_ESM.pdf]

Whole genome sequence and characterisation of *Streptococcus suis* 3112, isolated from snakeskin gourami, *Trichopodus pectoralis*

Running Title

Whole genome sequence and characterisation of *Streptococcus suis* 3112

Authors

Pakorn Aiewsakun[1,2]*, Wuthiwat Ruangchai[2], Bharkbhoom Jaemsai[1,2], Thavin Bodharamik[2], Watcharachai Meemetta[3], Saengchan Senapin[3,4]

Author affiliations

1. Department of Microbiology, Faculty of Science, Mahidol University, Bangkok, Thailand 10400

2. Pornchai Matangkasombut Center for Microbial Genomics, Department of Microbiology, Faculty of Science, Mahidol University, Bangkok, Thailand 10400

3. Fish Health Platform, Center of Excellence for Shrimp Molecular Biology and Biotechnology (Centex Shrimp), Faculty of Science, Mahidol University, Bangkok, 10400, Thailand.

4. National Center for Genetic Engineering and Biotechnology (BIOTEC), National Science and Technology Development Agency (NSTDA), Pathum Thani, 12120, Thailand

*Corresponding author contact

Pakorn Aiewsakun: Department of Microbiology, Faculty of Science, Mahidol University, 272, Rama VI Road, Ratchathewi, Bangkok, 10400, Thailand; +66 2 201 5543; pakorn.aie@mahidol.ac.th

Supplementary information

Supplementary Figures

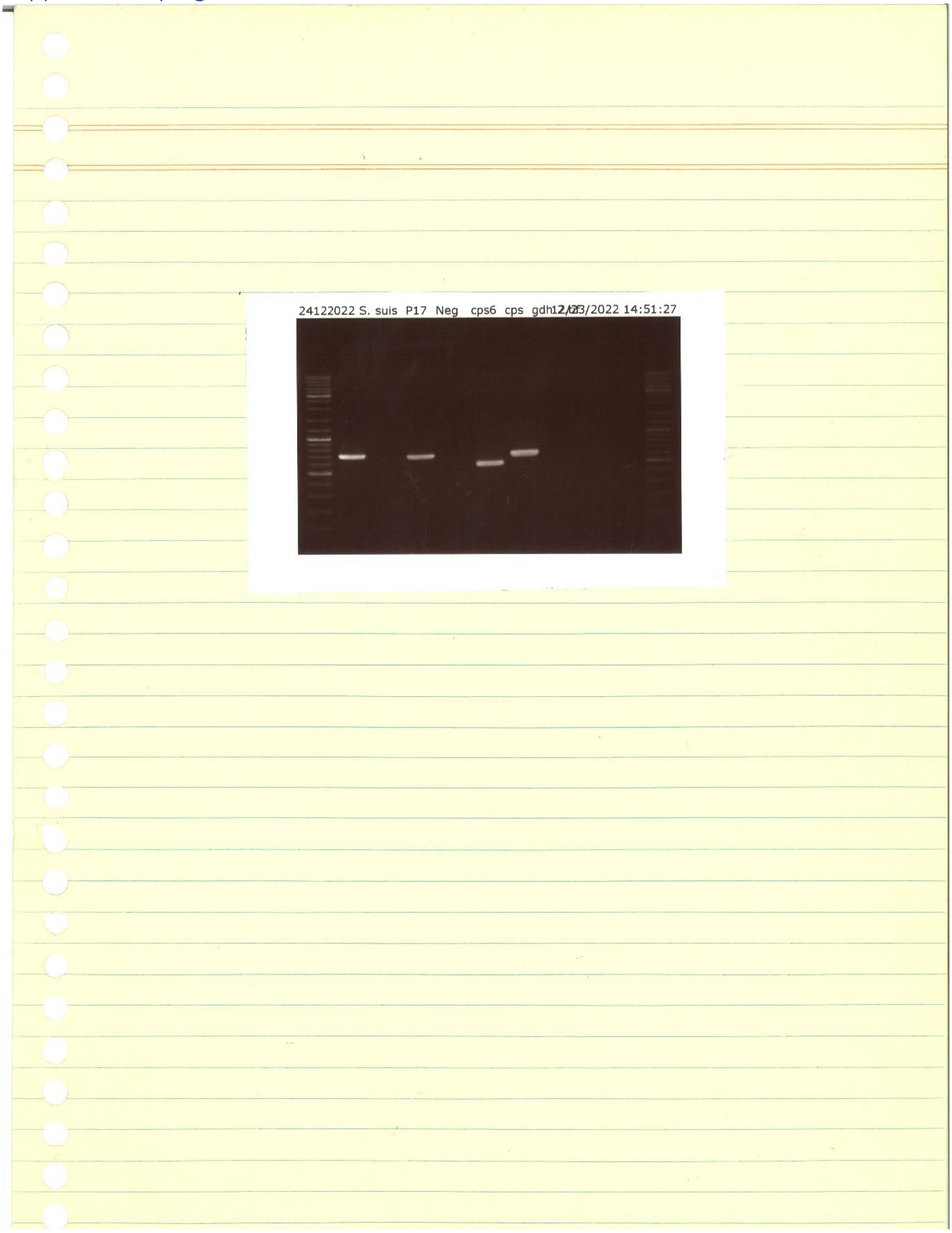

Supplementary Figure 1. Direct printout of the gel image shown in Figure 2A obtained from the Gel Documentation Systems (Aplegen, USA) machine.

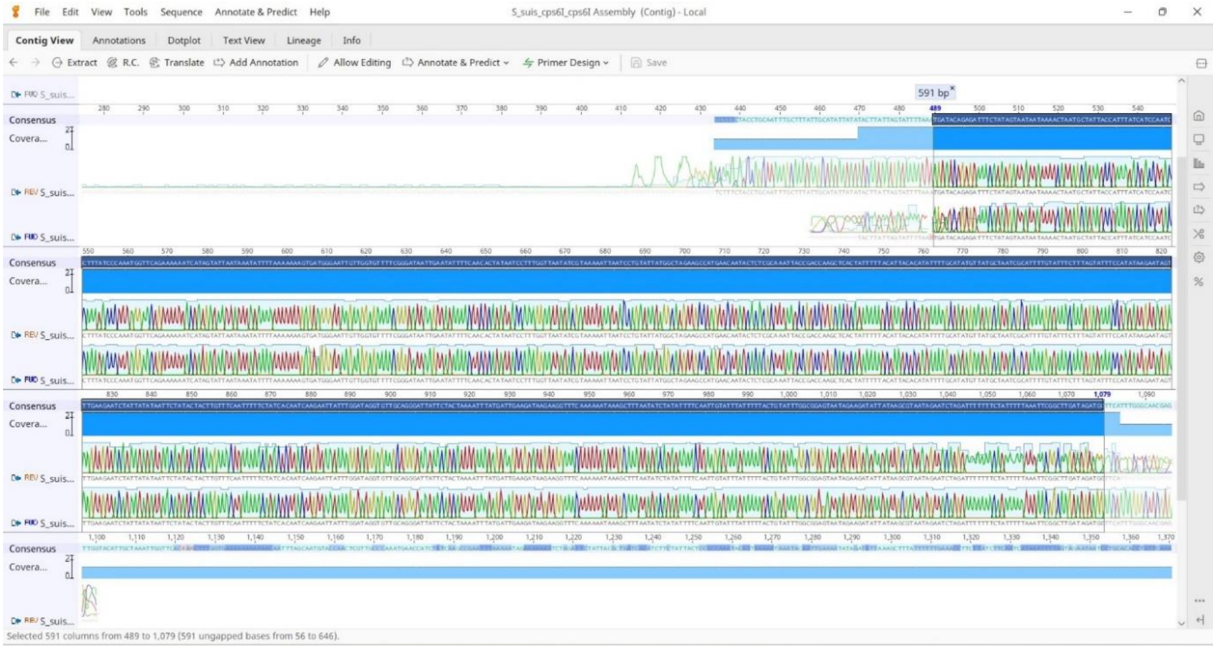

Supplementary Figure 2. Consensus sequence analysis of the bi-directional reads of the *cps6I* gene fragment generated by Sanger sequencing (591 bp). The sequence obtained is identical to the one within the assembled genome (**Supplementary Table 7**).

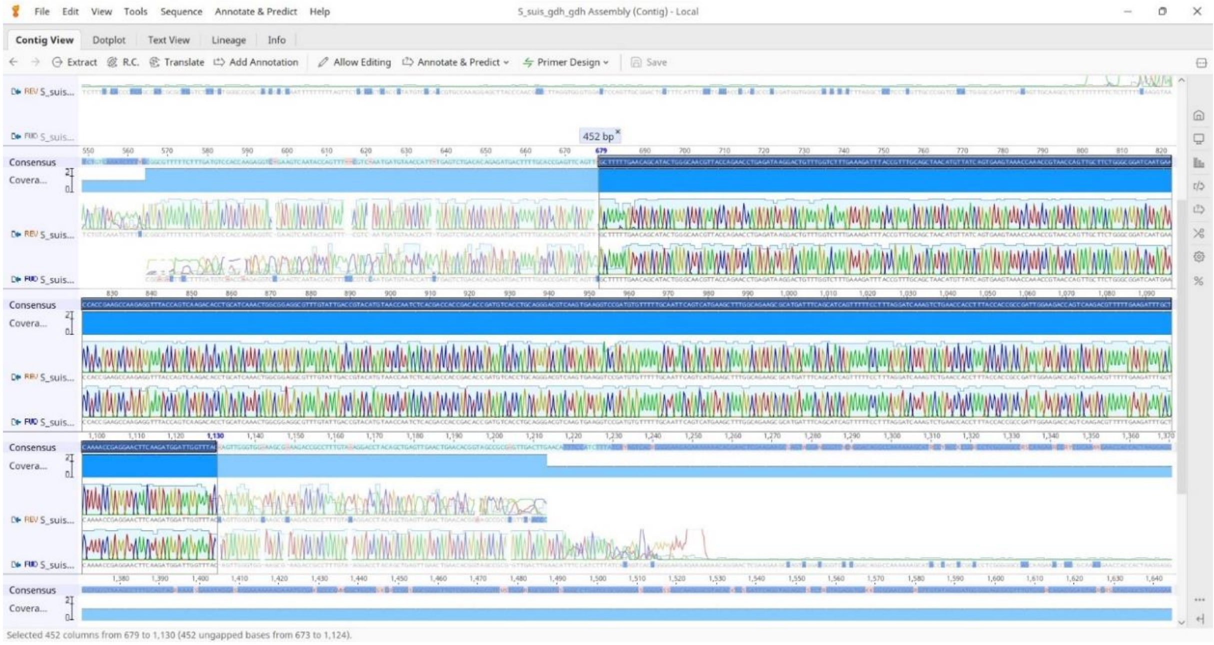

Supplementary Figure 3. Consensus sequence analysis of the bi-directional reads of the *gdh* gene fragment generated by Sanger sequencing (452 bp). The sequence obtained is identical to the one within the assembled genome (**Supplementary Table 7**).

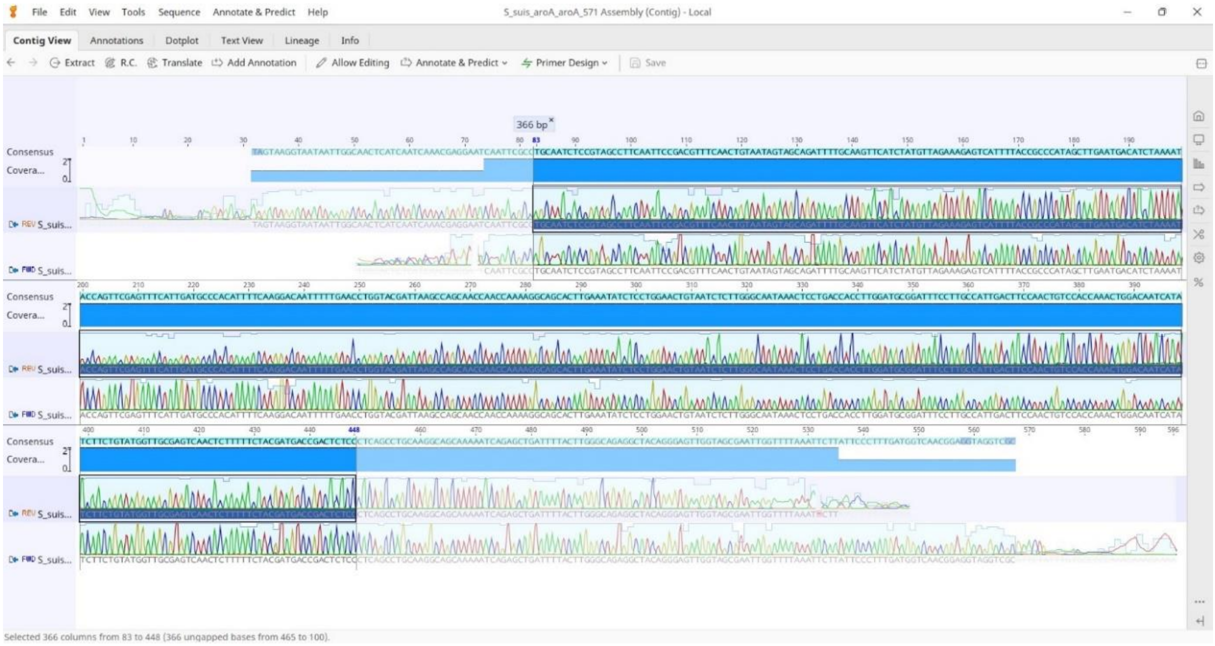

Supplementary Figure 4. Consensus sequence analysis of the bi-directional reads of the *aroA* gene fragment generated by Sanger sequencing (366 bp). The sequence obtained is identical to the one within the assembled genome (**Supplementary Table 7**).

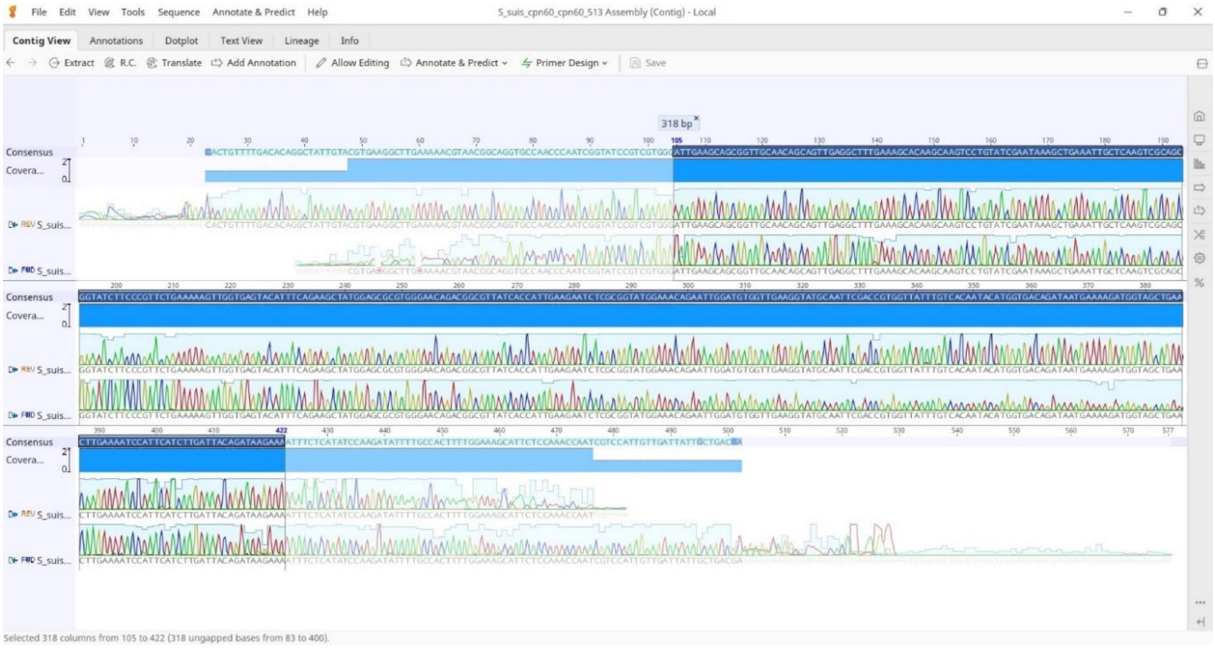

Supplementary Figure 5. Consensus sequence analysis of the bi-directional reads of the *cpn60* gene fragment generated by Sanger sequencing (318 bp). The sequence obtained is identical to the one within the assembled genome (**Supplementary Table 7**).

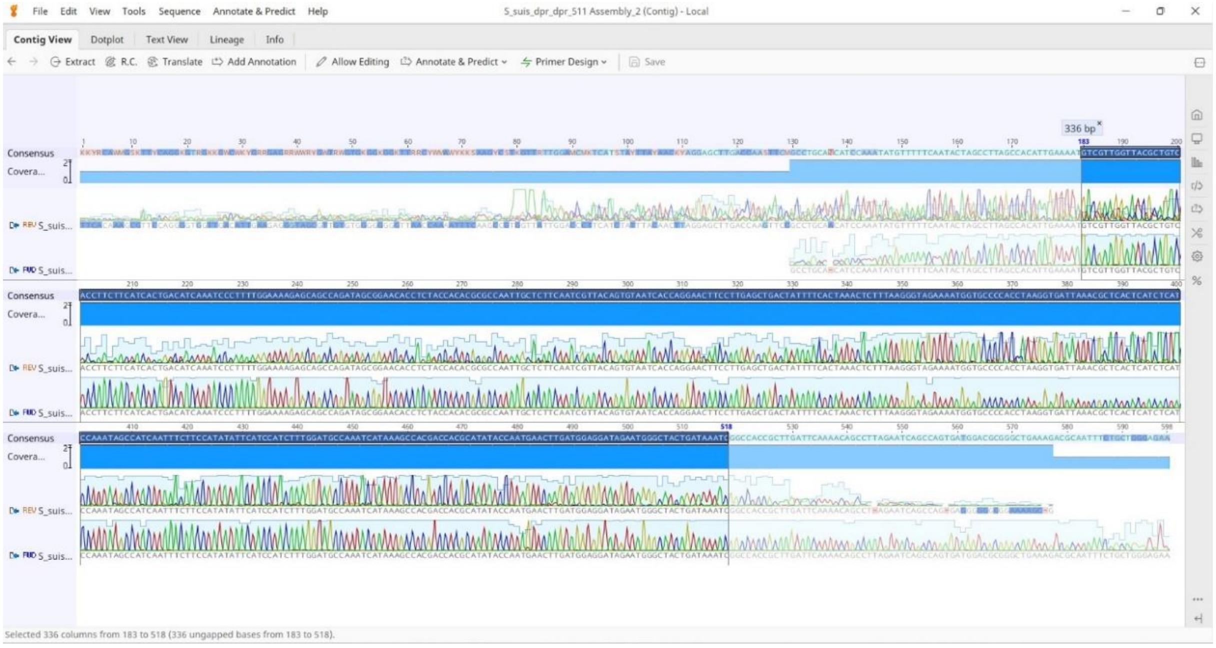

Supplementary Figure 6. Consensus sequence analysis of the bi-directional reads of the *dpr* gene fragment generated by Sanger sequencing (336 bp). The sequence obtained is identical to the one within the assembled genome (**Supplementary Table 7**).

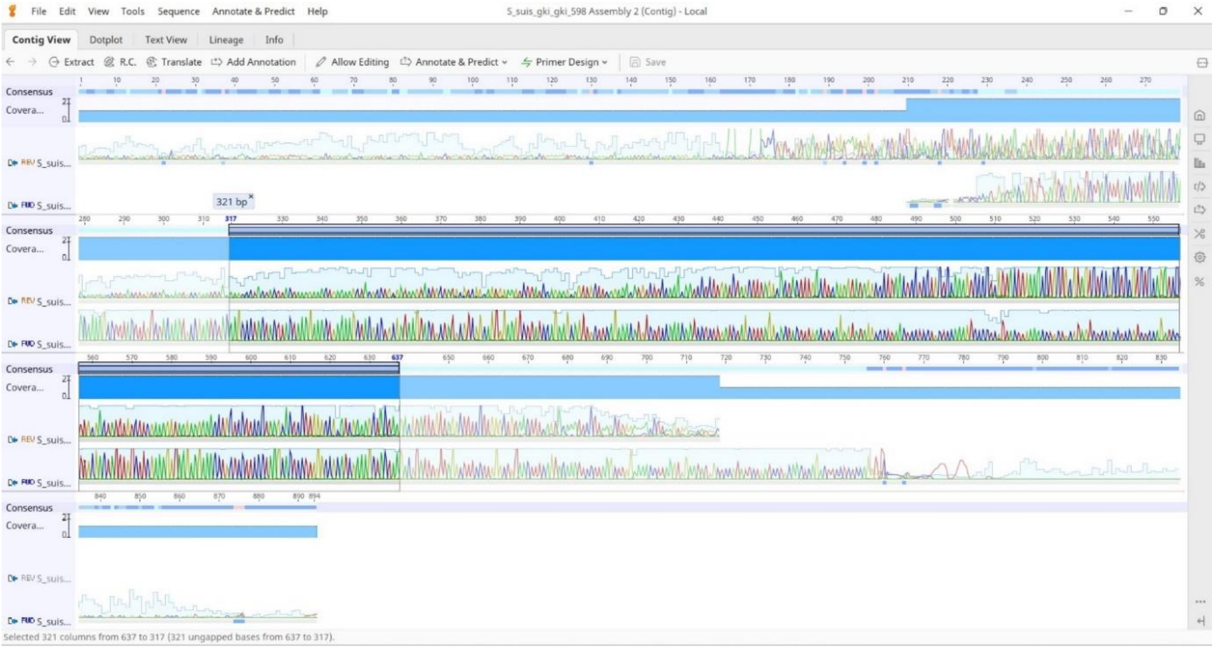

Supplementary Figure 7. Consensus sequence analysis of the bi-directional reads of the *gki* gene fragment generated by Sanger sequencing (321 bp). The sequence obtained is identical to the one within the assembled genome (**Supplementary Table 7**).

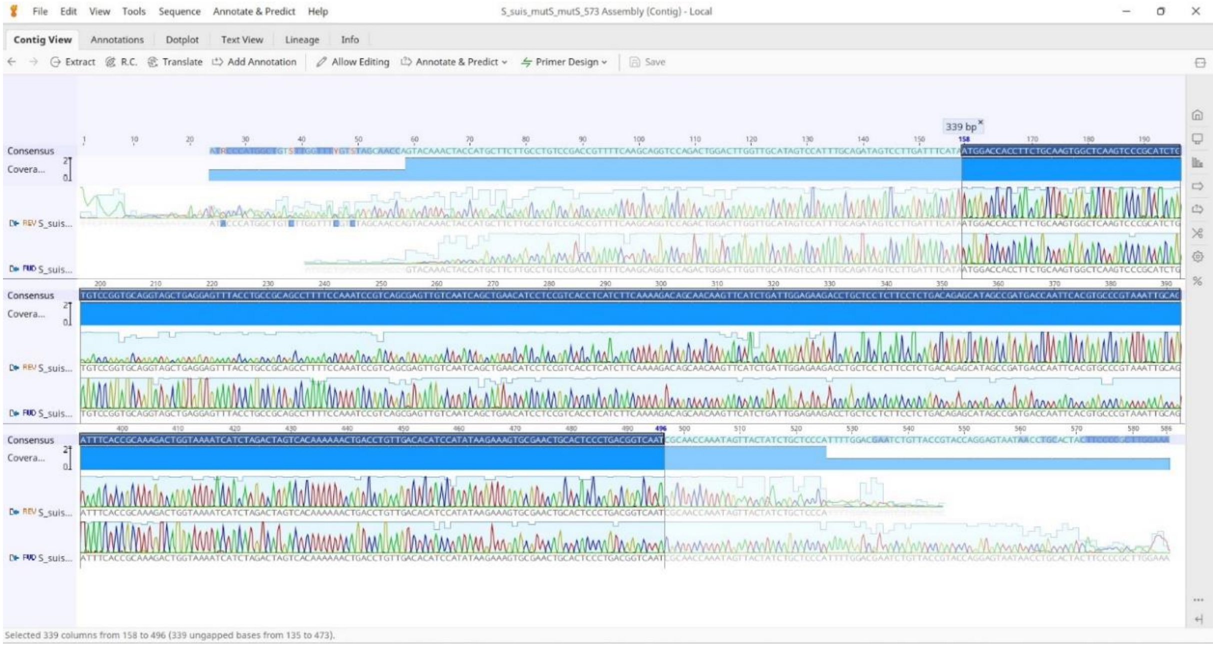

Supplementary Figure 8. Consensus sequence analysis of the bi-directional reads of the *mutS* gene fragment generated by Sanger sequencing (339 bp). The sequence obtained is identical to the one within the assembled genome (**Supplementary Table 7**).

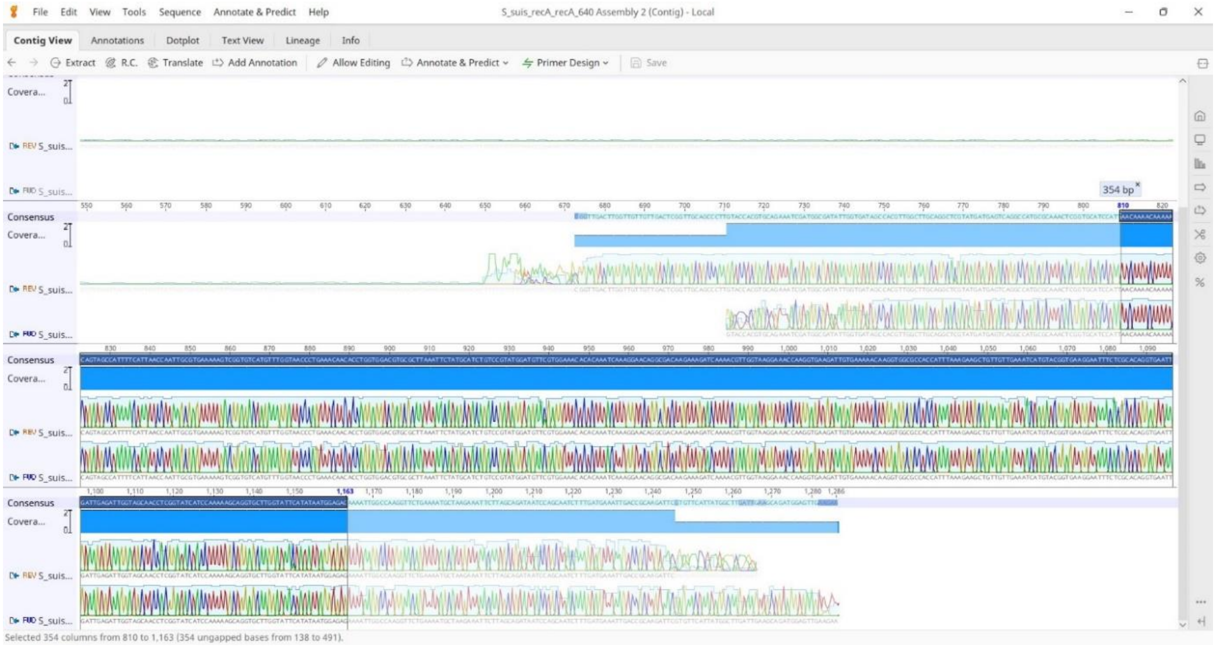

Supplementary Figure 9. Consensus sequence analysis of the bi-directional reads of the *recA* gene fragment generated by Sanger sequencing (354 bp). The sequence obtained is identical to the one within the assembled genome (**Supplementary Table 7**).

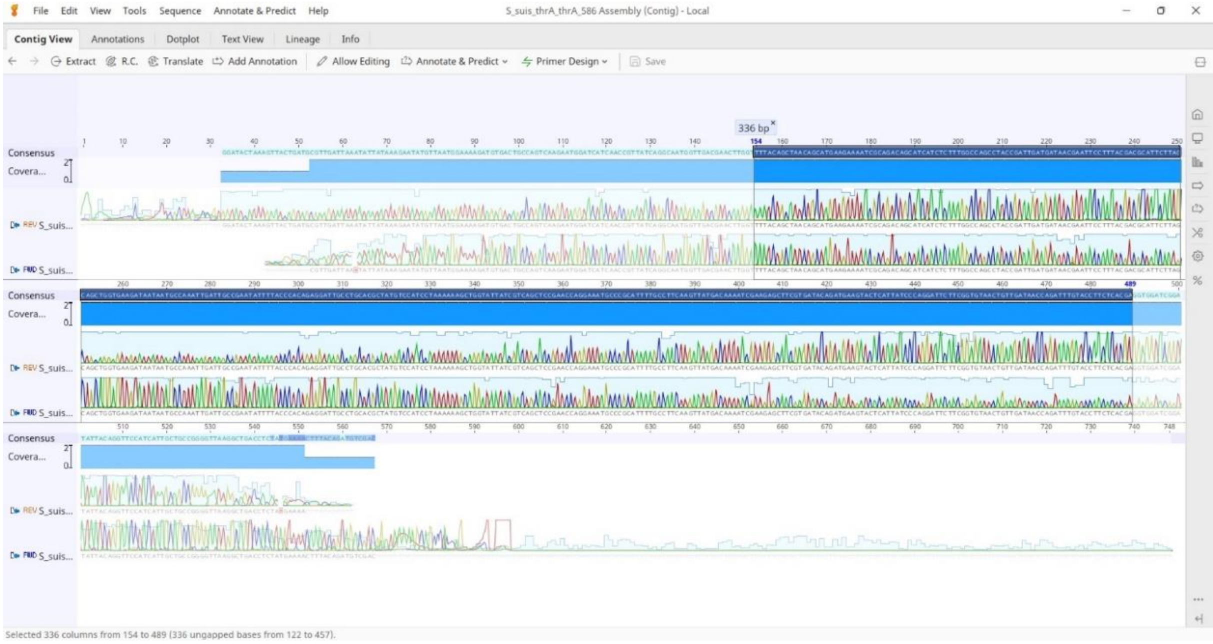

Supplementary Figure 10. Consensus sequence analysis of the bi-directional reads of the *thrA* gene fragment generated by Sanger sequencing (336 bp). The sequence obtained is identical to the one within the assembled genome (**Supplementary Table 7**).

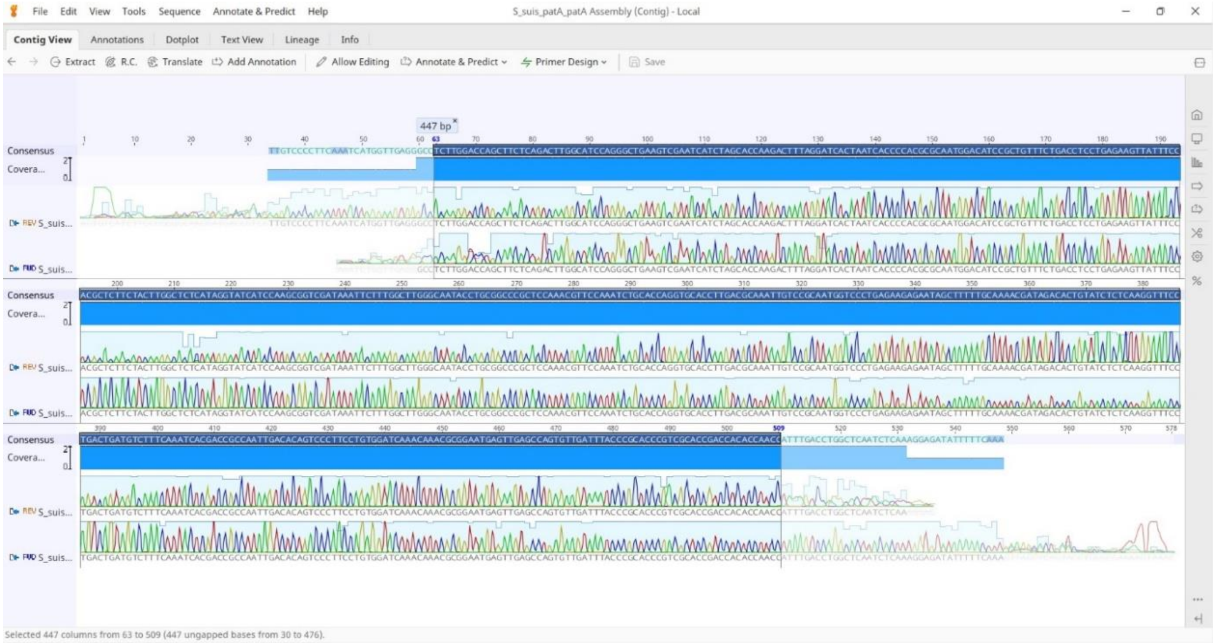

Supplementary Figure 11. Consensus sequence analysis of the bi-directional reads of the *patA* gene fragment generated by Sanger sequencing (447 bp). The sequence obtained is identical to the one within the assembled genome (**Supplementary Table 7**).

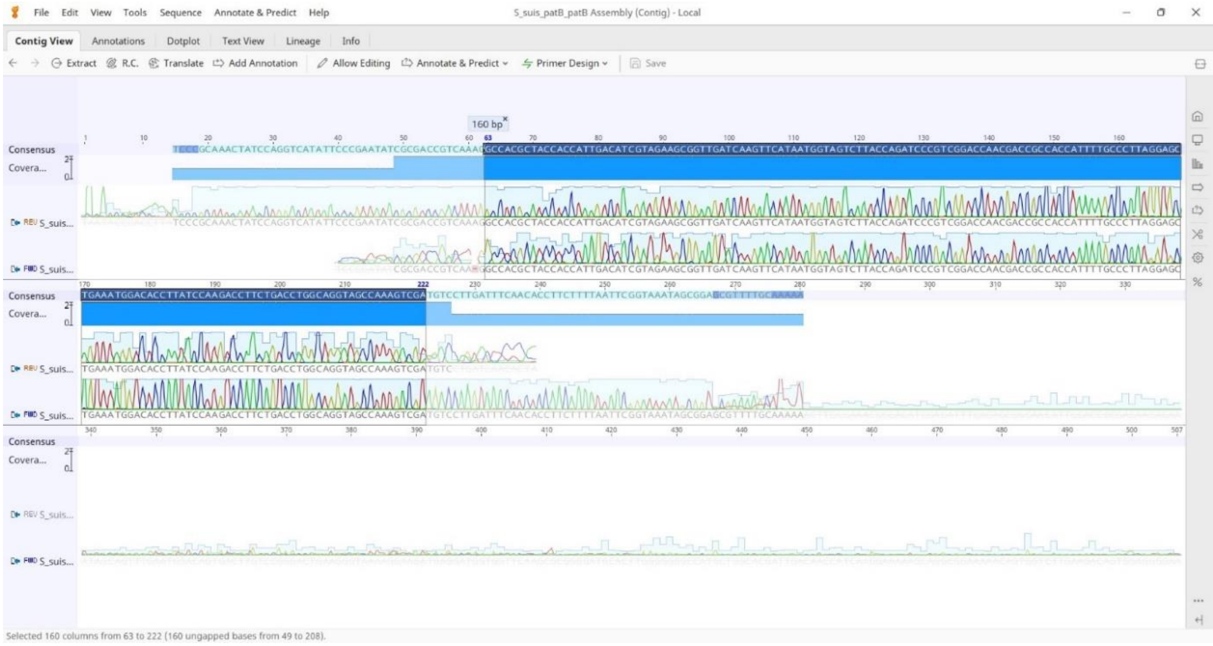

Supplementary Figure 12. Consensus sequence analysis of the bi-directional reads of the *patB* gene fragment generated by Sanger sequencing (160 bp). The sequence obtained is identical to the one within the assembled genome (**Supplementary Table 7**).

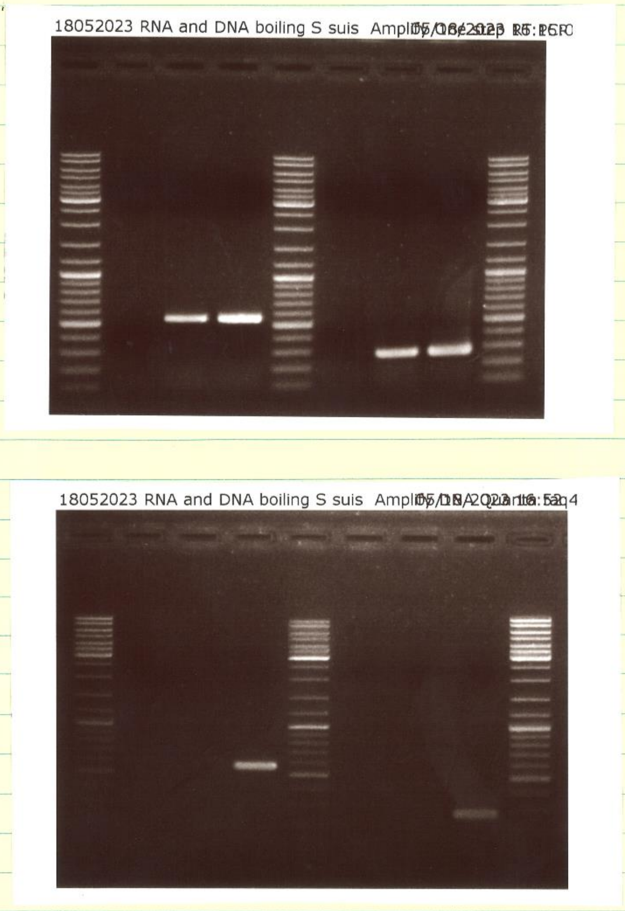

Supplementary Figure 13. Direct printouts of the gel images shown in Figure 4 obtained from the Gel Documentation Systems (Aplegen, USA) machine.

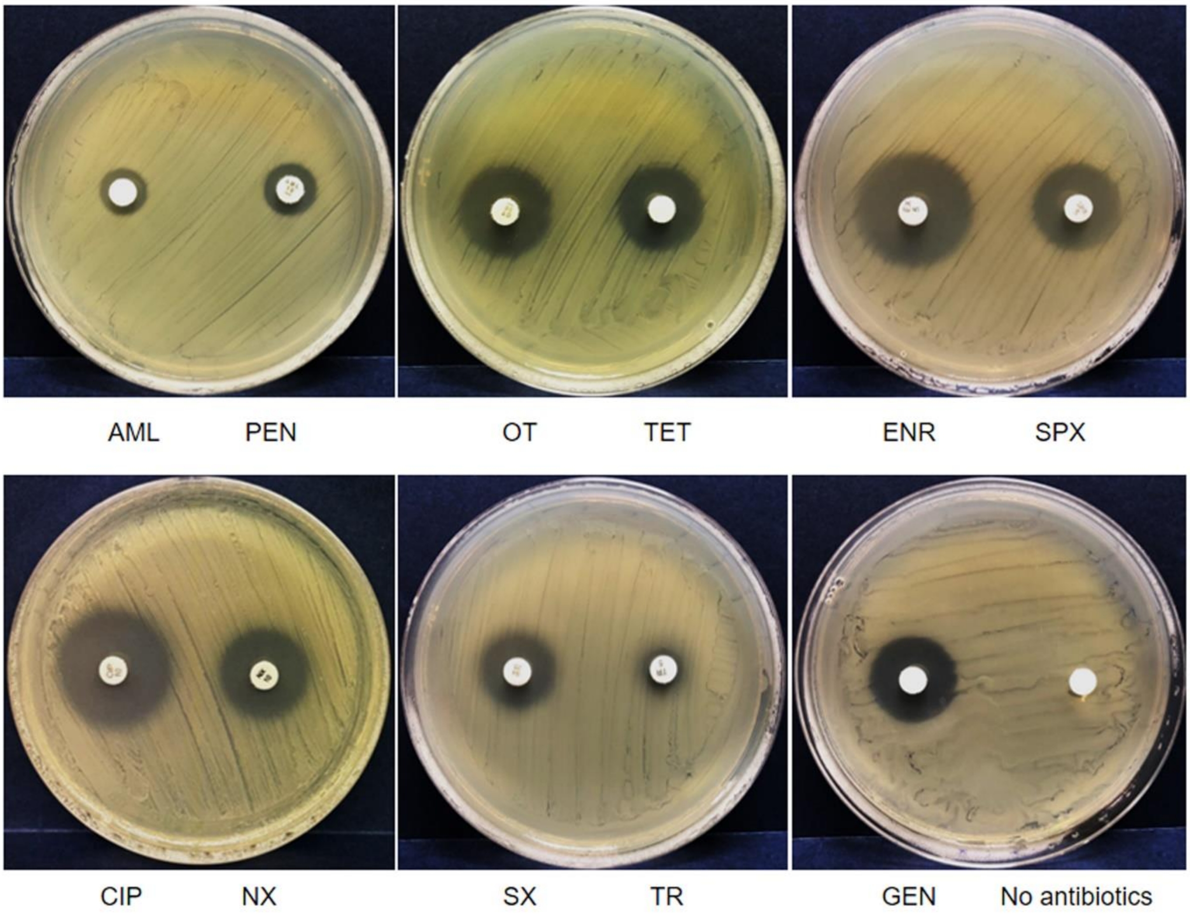

Supplementary Figure 14. One replicate of disk diffusion assays assessing the susceptibility of *Staphylococcus aureus* 1020 to eleven antimicrobial drugs from five distinct classes. The tested drugs included β-lactams (AML: amoxicillin, and PEN: penicillin), tetracyclines (OT: oxytetracycline, and TET: tetracycline), fluoroquinolones (ENR: enrofloxacin, SPX: sparfloxacin, CIP: ciprofloxacin, and NX: norfloxacin), sulphonamides (SX: sulphamethoxazole, and TR: trimethoprim), and an aminoglycoside (GEN: gentamicin). See **Supplementary Table 6** for detailed antimicrobial susceptibility testing results.

Supplementary Tables

Supplementary Table 1. Virulence factors detected in *S. suis* 3112 by VFanalyzer

Supplementary Table 2. Mobile genetic elements detected in *S. suis* 3112 by MobileOG

Supplementary Table 3. Contextualising *S. suis* genomes in the phylogenetic analysis.

Supplementary Table 4. Whole genome comparison between *S. suis* 3112 against BAPS6 and *S. suis* 2524 isolates.

Supplementary Table 5. BAPS6 members' serotypes.

Supplementary Table 6. Antimicrobial susceptibility testing results for *Staphylococcus aureus* 1020, performed as control. Disk diffusion assays were conducted to evaluate the susceptibility of *Staphylococcus aureus* 1020 to eleven antimicrobial drugs from five distinct classes. The experiment was performed in triplicate, and the mean diameters of the inhibition zones (mm) are reported with standard deviations. Zone diameter interpretive standard chart is provided in the table. Images of one replicate of the disk diffusion assays are shown in **Supplementary Figure 14**.

Supplementary Table 7. Comparison between the gene sequences obtained from Sanger sequencing and those in the assembled *S. suis* 3112 genome (CP097577.2)

Supplementary Data

Supplementary Data 1. Alignment used in the phylogenetic analysis in FASTA format.

Supplementary Data 2. Phylogenetic network shown in Figure 3A in NEXUS format.
